# Supraspinal, spinal, and motor unit adjustments to fatiguing isometric contractions of the knee extensors at low and high submaximal intensities in males

**DOI:** 10.1152/japplphysiol.00675.2023

**Published:** 2024-05-02

**Authors:** Luca Angius, Alessandro Del Vecchio, Stuart Goodall, Kevin Thomas, Paul Ansdell, Elliot Atkinson, Dario Farina, Glyn Howatson

**Affiliations:** ^1^Department of Sport, Exercise and Rehabilitation, Faculty of Health and Life Sciences, Northumbria University, Newcastle upon Tyne, United Kingdom; ^2^Department of Bioengineering, Imperial College London, London, United Kingdom; ^3^Department of Artificial Intelligence in Biomedical Engineering, Friedrich-Alexander-University Erlangen-Nuremberg, Erlangen, Germany; ^4^Water Research Group, North-West University, Potchefstroom, South Africa

**Keywords:** exercise, fatigue, high-density EMG, motor unit, transcranial magnetic stimulation

## Abstract

Contraction intensity is a key factor determining the development of muscle fatigue, and it has been shown to induce distinct changes along the motor pathway. The role of cortical and spinal inputs that regulate motor unit (MU) behavior during fatiguing contractions is poorly understood. We studied the cortical, spinal, and neuromuscular response to sustained fatiguing isometric tasks performed at 20% and 70% of the maximum isometric voluntary contraction (MVC), together with MU behavior of knee extensors in healthy active males. Neuromuscular function was assessed before and after performance of both tasks. Cortical and spinal responses during exercise were measured via stimulation of the motor cortex and spinal cord. High-density electromyography was used to record individual MUs from the vastus lateralis (VL). Exercise at 70%MVC induced greater decline in MVC (*P* = 0.023) and potentiated twitch force compared with 20%MVC (*P* < 0.001), with no difference in voluntary activation (*P* = 0.514). Throughout exercise, corticospinal responses were greater during the 20%MVC task (*P* < 0.001), and spinal responses increased over time in both tasks (*P* ≤ 0.042). MU discharge rate increased similarly after both tasks (*P* ≤ 0.043), whereas recruitment and derecruitment thresholds were unaffected (*P* ≥ 0.295). These results suggest that increased excitability of cortical and spinal inputs might be responsible for the increase in MU discharge rate. The increase in evoked responses together with the higher MU discharge rate might be required to compensate for peripheral adjustments to sustain fatiguing contractions at different intensities.

**NEW & NOTEWORTHY** Changes in central nervous system and muscle function occur in response to fatiguing exercise and are specific to exercise intensity. This study measured corticospinal, neuromuscular, and motor unit behavior to fatiguing isometric tasks performed at different intensities. Both tasks increased corticospinal excitability and motor unit discharge rate. Our findings suggest that these acute adjustments are required to compensate for the exercise-induced decrements in neuromuscular function caused by fatiguing tasks.

## Graphical abstract

**Figure:**
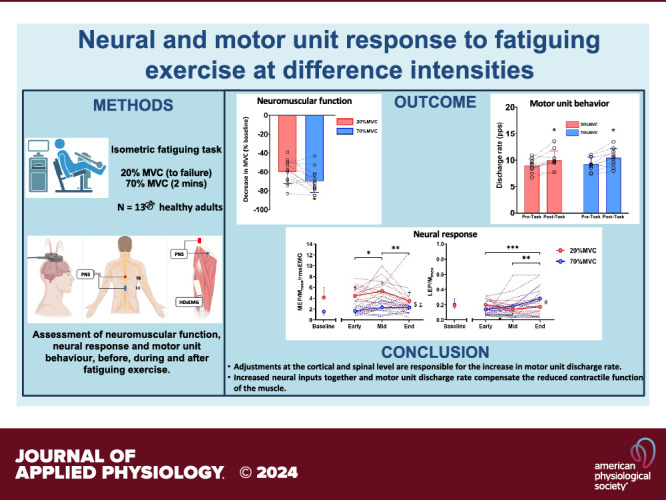

## INTRODUCTION

During sustained maximal and submaximal contractions, the ability to voluntarily generate force progressively declines despite maximal effort being exerted (1, 2), a phenomenon commonly ascribed to adjustments in neuromuscular function, the etiology of which is determined by the intensity (and consequent duration) of the muscle action (2).

A wide variety of physiological processes along the motor pathway are possible sites for decrements in neuromuscular function (3, 4). Many of these processes are inferred from measurements of central or peripheral function after voluntary and involuntary muscle actions. During low-intensity tasks, generally performed below 30% of maximal voluntary contraction (MVC), significant central nervous system impairments have been inferred from reductions in voluntary activation (VA) (5, 6). Conversely, contractile dysfunction seems to play a more prominent role when higher-intensity tasks are performed (1, 7).

Maximal and submaximal fatiguing contractions are also associated with disparate adjustments in the surface electromyographic (EMG) response during fatiguing exercise (8). Despite many investigations drawing conclusions from surface EMG data measured with bipolar configurations, the precise changes in motor unit behavior are not well understood. High-density surface EMG (HDsEMG) allows for decomposition analysis, which enables samples of motor units (MUs) to be identified. The information related to changes in motor unit behavior can provide important knowledge on how the nervous system controls muscle force generation during fatiguing contractions (8), but as yet this area of study is limited.

Motor unit behavior reflects the transformation of synaptic input from descending tracts and sensory feedback (9). Considering that varying intensities of exercise elicit distinct neurophysiological adjustments (10–13), it is plausible that variations in exercise intensity will have differential effects on motor unit behavior. A recent study by Martinez-Valdes et al. (14) suggested that future studies should combine motor unit recordings with noninvasive neurostimulation during sustained contractions until failure, thereby enabling a greater understanding of the relationship between the etiology of decrements in neuromuscular function and motor unit behavior. To date, experimental works have investigated the effect of exercise performed to task failure at different intensities on motor unit behavior of the tibialis anterior (15) and knee extensors (16). However, no measures of neuromuscular function or neural responses were included. As such, the current knowledge concerning the exercise-induced decline in neuromuscular function, and acute changes in neural response on motor unit behavior, are currently unknown. The assessment of the neuromuscular function and neural responses is important to gain a better understanding of to what extent these factors might influence MU behavior.

Given the limited information in this field, we aimed to further extend the current knowledge by assessing the neuromuscular function at task failure and neural responses during exercise at different sites of the motor pathway by means of magnetic stimulation (TMS), electrical stimulation of the spinal cord, and percutaneous stimulation of the femoral nerve. Therefore, this investigation aimed to quantify central and peripheral contributors to decrements in neuromuscular function during and after fatiguing isometric exercise of the knee extensors at disparate intensities and the behavior of the vastus lateralis motor units after exercise in a group of healthy males. We combined neurostimulation techniques and HDsEMG to study the effect of fatiguing muscular contractions of the knee extensors at lower and higher intensities. It was hypothesized that the locus of adjustments in neuromuscular function would change in line with the intensity of exercise and such changes would be accompanied by disparate alterations in motor unit behavior.

## MATERIALS AND METHODS

### Ethical Approval

The study received institutional ethical approval from Northumbria University Health and Life Sciences Research Ethics Committee (submission ref: 17182) and was conducted in line with guidance set out in the Declaration of Helsinki. Before any experimental procedures, volunteers provided written informed consent to participate in the study.

### Participants

A group of 13 recreationally active white Caucasian males [mean ± SD age 32.1 ± 7.3 yr; stature 172.5 ± 22.2 cm; mass 81.5 ± 13.7 kg; body mass index (BMI) 25.6 ± 3.9 kg/m^2^] from the staff and postgraduate community at Northumbria University volunteered to participate. All participants were physically active, had no history of cardiorespiratory or neurological disease, and were not injured or taking medication at the time of the study. Participants were instructed to refrain from alcohol, caffeine consumption, and strenuous lower body physical activity (48 h) before experimental visits.

### Experimental Design

**Figure 1.**
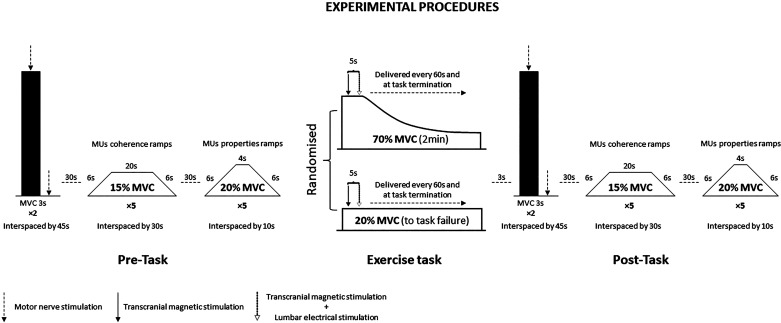
Overview of the experimental design and the experimental procedures performed. MU, motor unit; MVC, maximal voluntary contraction.

All participants visited the laboratory three times, completing familiarization and two experimental trials over a 2-wk period. The experimental visits were separated by a minimum of 48 h, allowing for recovery (17), and were presented in a randomized crossover design. The time of day for each testing session remained consistent (±1 h) to limit diurnal variations in maximal force-generating capacity and corticospinal excitability (18). After a thorough familiarization with the methods used to assess neuromuscular function, two fatiguing trials were performed with the dominant knee extensors (19). To understand motor unit behavior at disparate intensities, the fatiguing tasks were conducted at a lower (20%MVC) and a higher (70%MVC) contraction intensity (see below). The two intensities were chosen to induce different adjustments at both central and peripheral levels as previously described (1, 2, 5–7). The experimental design is illustrated in Fig. 1.

### Experimental Procedures

#### Neuromuscular function assessments.

Neuromuscular function was assessed before and after exercise, starting immediately at task failure. Preexercise neuromuscular function assessment began with a standardized warm‐up (2 × contractions at 25%, 50%, and 75% of perceived maximal effort), and then two MVCs interspaced by 45 s were performed. The highest MVC was used to set the contraction intensity for the fatiguing task and series of ramped contraction on each session. The ramped contractions were performed to measure aspects of motor unit behavior before and after the postexercise MVC after task failure. Five ramp contractions were performed at 15% MVC with rate of force development of 4% MVC·s^−1^ with a plateau phase of 20 s, and five were performed at 20% MVC, whereby the rate of force development was 3.3% MVC·s^−1^ with a plateau phase of 4 s. The 15%MVC ramps were interspaced by 30 s, whereas the 20%MVC ramps were interspaced by 10 s. All ramps were performed at the same absolute intensity, based on the preexercise MVC. After task failure in both conditions, the first ramp was performed within 30 s after the postexercise MVC.

Thereafter, two further (∼3 s) MVCs were performed, separated by 30 s. During the second of these MVCs, motor nerve stimulation (MNS) was delivered during the plateau of peak force, and ∼2 s after, to quantify voluntary activation (20), the quadriceps potentiated twitch amplitude (Q_tw.pot_), and maximal motor response (M_max_). Single-pulse TMS was subsequently delivered during three contractions at 20% or 70% MVC, and a further three contractions at the same intensity were performed with lumbar stimulation superimposed (see below). Ten-second rest was given between contractions. Measures of neuromuscular function (MVC, Q_tw.pot_, and VA) were measured immediately at task failure, before the postexercise ramp contractions. MVC, Q_tw.pot_, and VA at postexercise were measured within 2 s after task termination while the first trapezoidal ramp was performed within 30 s. The experimental procedures are illustrated in Fig. 1.

#### Transcranial magnetic stimulation.

Single stimuli (1-ms duration) were delivered to the contralateral motor cortex via a concave double-cone coil oriented to induce a posterior-to-anterior intracranial current (110-mm diameter, maximum output 1.4 T), powered by a monopulse stimulator (Magstim^200^, The Magstim Company, Whitland, UK). Optimal coil placement was determined as the position that elicited the greatest vastus lateralis (VL) motor evoked potential (MEP) during a 10% MVC using 50–70% stimulator output. This position was marked on the scalp with indelible ink to ensure consistent placement during trials. Active motor threshold (aMT) was determined as the stimulator intensity that elicited a MEP of >200 µV in three out of five stimulations during a 10% MVC contraction. Stimulator intensity was increased in 5% steps from 35% of stimulator output until a consistent MEP was found. Thereafter, stimulus intensity was reduced in 1% steps until the lowest intensity to elicit a MEP of >200 μV was found. Mean aMT was not different between visits (47 ± 10% vs. 41 ± 8%, *P* = 0.119). At baseline, corticospinal excitability was determined in the three 20% or 70% MVC contractions with a test pulse of 120% aMT.

#### Lumbar electrical stimulation.

To assess spinal excitability/responsiveness, lumbar evoked potentials (LEPs) were elicited with a constant-current stimulator (1-ms pulse duration; DS7AH, Digitimer Ltd, Welwyn Garden City, UK). The cathode was centered over the first lumbar spinous process (5 × 9 cm; Nidd Valley Medical Ltd., Bordon, UK) with the electrode aligned to the center of the vertebral column. The surface area of the cathode covered two spinous processes above and below the center point (T11–L3). A cathode of large area was chosen as it produced less discomfort and greater tolerance by participants (21, 22). The anode (2.5 cm^2^) was placed 5 cm above the upper edge of the cathode (22), corresponding to the level of the eighth thoracic spinous process (T8), as this stimulating site has recently been shown to activate corticospinal axons at the level of lumbar spinal segments (23). The preexercise LEP was standardized to elicit 15–25% of M_max_ during contractions at 20% and 70% MVC. Lumbar stimulation was delivered 100 ms into a 200-ms silent period (SP; conditioned) to quantify excitability of the spinal cord without the presence of background neural drive (24). For these conditioned LEPs (SP-LEPs), the TMS intensity to produce a SP of 200 ms was not different between visits (57 ± 8% vs. 59 ± 11%, *P* = 0.731); likewise the intensity of subsequent lumbar stimulation was similar (185 ± 62 vs. 181 ± 56 mA, *P* = 0.291).

#### Motor nerve stimulation.

Single electrical stimuli (200-µs duration) were delivered to the femoral nerve of the exercising limb with a constant-current stimulator (DS7AH, Digitimer Ltd) via adhesive surface electrodes (CF3200, Nidd Valley Medical Ltd., Harrogate, UK). The cathode was placed over the nerve, high in the femoral triangle, in the position that elicited the greatest twitch amplitude (Q_tw_) and M_wave_ in the VL at rest. The anode was placed halfway between the greater trochanter and iliac crest. Stimulation intensity was increased in 20-mA stepwise increments until the highest Q_tw_ and M_max_ were elicited. Then, stimulation intensity was increased by 30% to ensure that supramaximal stimulation was delivered. Mean stimulus intensity was not different between visits (241 ± 41 vs. 249 ± 47 mA, *P* = 0.388).

#### Force and bipolar electromyography.

During assessments of neuromuscular function, participants sat on a custom-built chair with knee and hip angles kept constant (both 90° flexion). A calibrated load cell (MuscleLab Force Sensor 300, Ergotest technology, Norway) was attached via a noncompliant cuff positioned 2 cm superior to the ankle malleoli on the participants’ dominant leg, to measure knee extensor force (N). Bipolar electromyographic signals were recorded continuously throughout both trials with surface electrodes (Ag/AgCl; Kendall H87PG/F, Covidien, Mansfield, MA). Electrodes were placed over the dominant VL, just above the HDsEMG array, consistent with surface EMG for noninvasive assessment of muscles (SENIAM) guidelines (25), and were connected to an EMG system (CED 1902, Cambridge Electronic Design, Cambridge, UK). Before placement of electrodes, the skin contact area was shaved, abraded, and cleaned with a 70% IPA alcohol wipe (FastAid, Robinson Healthcare, Worksop, UK). Signal recordings obtained from the bipolar EMG electrodes were used to determine TMS, LEP, and motor nerve stimulation parameters in both experimental sessions. The same recordings were also used to obtain information related to cortical, spinal, and electrical muscle response and variations in EMG activity during the fatiguing task in both visits. Signals were amplified [gain ×100 for EMG and ×300 for force (CED 1902)], band-pass filtered (EMG only: 20–1,000 Hz), digitized (EMG: 2 kHz; force: 5 kHz; CED 1401, Cambridge Electronic Design), and analyzed offline (Spike2 v8, Cambridge Electronic Design).

#### High-density electromyographic recordings.

Electrical activity of the VL was recorded by means of HDsEMG using a single adhesive array of 64 equally spaced electrodes (13 rows × 5 columns; gold coated; diameter 1 mm; interelectrode distance 8 mm; OT Bioelettronica, Turin, Italy). The array was moved in the distal portion of each muscle to estimate the direction of muscle fibers that corresponded to the alignment of action potentials propagating along the array, without substantial changes in waveform shape (26). The HDsEMG signals were recorded in monopolar mode with a multichannel amplifier (3-dB bandwidth, 10–500 Hz; EMG‐Quattrocento, OT Bioelettronica). The EMG and force signals were concurrently sampled at 2,048 Hz with 16 bits/sample, amplified (×150), and band‐pass filtered. The signals were converted to digital data with a multichannel amplifier with 16-bit resolution (3-dB bandwidth, 20–500 Hz; EMG‐Quattrocento, OT Bioelettronica).

#### Fatiguing exercise.

After the completion of preexercise measurements, the fatiguing exercises were completed. The 20%MVC trial required a contraction to be held until task failure, deemed to be a 5% drop below the target force for a period longer than 5 s. Throughout the 20%MVC contraction, a single TMS and LEP response was evoked at 10 s and then every minute until task failure. During the 70%MVC trial, participants aimed to contract at the target level for 2 min or to continue contracting with maximal effort until 2 min if force dropped below 70% MVC. TMS and LEP responses were evoked at 10 s, 1 min, and 2 min. During both contractions, strong verbal encouragement was provided. Immediately upon task failure or task termination, MVC, VA, and Q_tw.pot_ were assessed before the postexercise ramp contractions.

### Data Analysis

#### Neuromuscular function analysis.

Voluntary activation was determined with the twitch interpolation technique [Merton (20)] by comparing the amplitude of the superimposed twitch (SIT) with the amplitude of the Q_tw.pot_ using the following formula: VA (%) = (1 − [SIT/Q_tw.pot_] × 100). At baseline and throughout fatiguing exercise, corticospinal (MEP/M_max_) and spinal (SP-LEPs) excitability were determined. To measure background muscle activity, the VL was recorded during the 50 ms before each stimulation or taken as the mean value during ramp contractions and normalized to baseline. To account for between-trial differences in background neural drive, evoked responses were further normalized to the prestimulus (100 ms) root mean square electromyography (rmsEMG) value (27). Data were compared at baseline and three points during exercise, Early (10 s), the midpoint (Mid; minute closest to the middle during 20%MVC and 1 min during 70%MVC), and at the end of exercise (End; task failure during 20%MVC and 2 min during 70%MVC), to allow for comparison between trials.

#### Motor unit properties analysis.

The HDsEMG signals were decomposed into single-motor unit activity by the convolutive blind source separation method (28). The identified motor units were analyzed by an operator (L.A.). Motor units were excluded when the pulse-to-noise ratio (PNR) was <30 dB (29). The recruitment and derecruitment thresholds [force value in % maximal voluntary force (MVF) corresponding to the first and last motor unit spike, respectively] and the average discharge rate of each motor unit were calculated from the discharge times identified after decomposition. The average discharge rate was calculated by considering the recruitment phase (i.e., the average of the first 4 action potentials), the steady phase (i.e., the average of the first 10 action potentials), and the derecruitment phase (i.e., the average of the last 4 action potentials). The input-output gain of the motor neuron pool was estimated by calculating the change in discharge rate during the steady phase relative to that at recruitment (ΔDR) as a function of the change in force from recruitment to the steady phase (ΔForce), as a function of the change in force from recruitment to the steady phase in percentage of the MVC performed at Pre-task (ΔForce_MVCpre-task_), and as a function of the change in force from recruitment to the steady phase in percentage of the corresponding MVC performed at Post-task (ΔForce_MVCcorr_).

#### Motor unit coherence analysis.

Motor unit coherence analysis was applied to the steady-state phase only during the long-duration ramps performed at 15% MVC. The coherence function, denoted as C*_xy_*(f), is calculated using the cross-spectrum and autospectral densities of the two signals, denoted as G*_xy_*(f), G*_xx_*(f), and G*_yy_*(f), respectively. The cross-spectrum represents the correlation between the two signals, whereas the autospectral densities capture the power of each individual signal. The coherence function is obtained by squaring the magnitude of the cross-spectrum and dividing it by the product of the spectral densities. To compute the coherence function, the data are divided into nonoverlapping windows of 1 s. The analysis was then performed separately on two groups of motor unit spike trains. The coherence bias, representing the maximum coherence value above the 100 Hz band, was estimated. It is important to note that the coherence function can reveal significant correlations at various frequency ranges within the sampling frequency resolution. In the adult spinal cord, three dominant peaks are commonly observed: a low-frequency range (0 to 5 Hz), a range around 5 to 12 Hz (tremor frequency), and the cortical beta band (15 to 30 Hz). These peaks represent the frequencies at which the motoneuronal fluctuations are synchronized or exhibit strong correlations.

### Statistical Analysis

Data are presented as means ± SD within the text and figures. Normal Gaussian distribution of data was confirmed with the Shapiro–Wilk test. The Greenhouse–Geisser correction to the degrees of freedom was applied when violation of sphericity was found. Data that were assessed at Pre-task and Post-task (change scores in parameters of neuromuscular function and motor unit coherence), along with the total impulse, were analyzed with a paired-samples *t* test. For variables assessed during exercise, a two-way (2 × 3) repeated-measures ANOVA was used to analyze differences between contraction intensity (20%MVC vs. 70%MVC) over time (Early, Mid, and End). For variables related to the motor unit properties and VL rmsEMG during steady-state phase of short- and long-duration ramps, a two-way (2 × 2) repeated-measures ANOVA was used to analyze differences between contraction intensity (20%MVC vs. 70%MVC) over time (Pre-task vs. Post-task). If significant main or interaction effects were observed, these were followed up with Tukey’s pairwise comparisons. The significance level for all statistical tests was set at *P* < 0.05 (IBM SPSS Statistics v28, New York, NY).

## RESULTS

### Exercise Performance

By the nature of the design, the 70%MVC task lasted 2 min and the exercise time for the 20%MVC task was 5 ± 2 min. Subsequently, the total impulse was greater in the 20%MVC trial compared with the 70%MVC trial (42 ± 12 vs. 34 ± 7 N·s; *P* = 0.017).

### Neuromuscular Function

**Figure 2.**
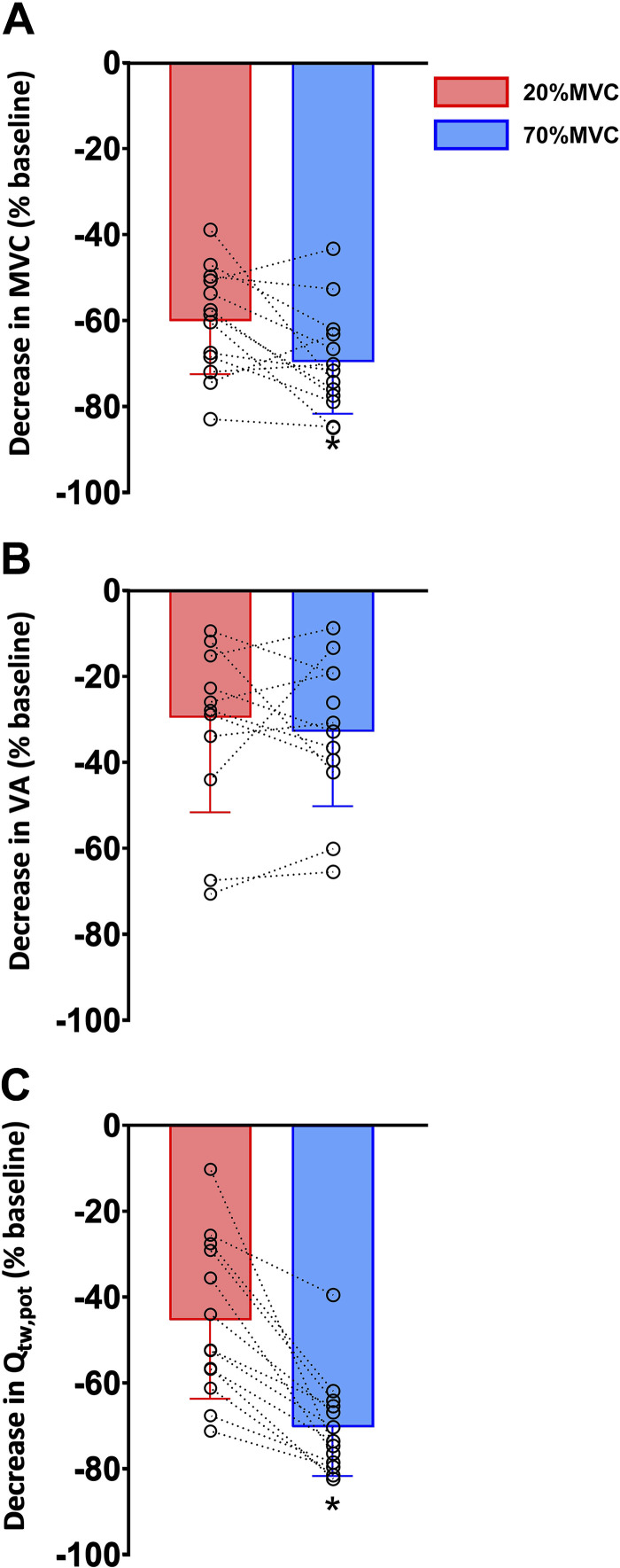
Neuromuscular function after both fatiguing tasks. Bar plots represent the average with data points representing individual data for maximum voluntary contraction (MVC, *A*), voluntary activation (VA) measured with motor nerve stimulation (*B*), and potentiated knee extensor twitch force (Q_tw_,_pot_, *C*). All parameters are expressed as decrease in percentage from baseline. *Significantly different from 20%MVC task (*P* < 0.05). Values are means ± SD for 13 participants.

**Figure 3.**
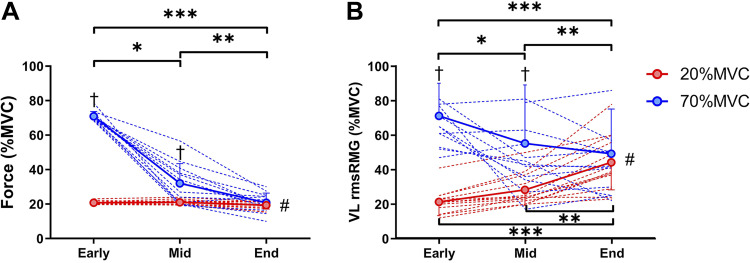
Force profile and electromyography activity of the vastus lateralis: force produced expressed as % of the maximal voluntary contraction (MVC) (*A*) and vastus lateralis root mean square electromyography activity (VL rmsEMG) expressed as % of the rmsEMG (*B*) throughout the 20%MVC (in red) and 70%MVC (in blue) tasks. #Condition × time interaction (*P* ≤ 0.001). †Significantly different from 20%MVC (*P* < 0.001). *Significantly different from Early to Mid (*P* < 0.05); **significantly different from Mid to End (*P* < 0.05); ***significantly different from Early to End (*P* < 0.05). Values are means ± SD for 13 participants.

Maximal voluntary force declined after performance of both tasks, with a greater reduction observed after 70%MVC (668 ± 125 to 203 ± 102 N, −70 ± 12%) versus 20%MVC (649 ± 125 to 253 ± 82 N, −60 ± 12%; *P* = 0.023) (Fig. 2*A*). The reduced MVC was accompanied by a reduction in Q_tw.pot_ in each task, with a greater contractile disturbance following the 70%MVC task (209 ± 41 to 64 ± 36 N, −70 ± 12% vs. 202 ± 40 to 110 ± 43 N, −45 ± 18%; *P* < 0.001; Fig. 2*C*). Voluntary activation was reduced after both tasks, with declines of similar magnitude for 20%MVC (93.1 ± 3.6 to 65.5 ± 20.4%, −30 ± 22%) and for 70%MVC (93.7 ± 3.2% to 63.1 ± 17.0%, −33 ± 17%; *P* = 0.514; Fig. 2*B*). The VL M_max_ was similar in each trial and remained unchanged after exercise (5.11 ± 2.75 to 4.94 ± 2.72 mV for 20%MVC and 4.81 ± 1.54 to 5.1 ± 1.74 mV for 70%MVC; *P* ≥ 0.382). The force produced (Fig. 3*A*) declined throughout 70%MVC from Early to End (*P* < 0.001) and was greater compared with 20%MVC at Early (70 ± 3% vs. 21 ± 1%; *P* < 0.001) and Mid (32 ± 11% vs. 21 ± 1%; *P* < 0.001), but it was similar at End (21 ± 6% vs. 19 ± 2%, *P* = 0.297). VL rmsEMG (Fig. 3*B*) was greater throughout 70%MVC compared with 20%MVC at Early and Mid (*P* ≤ 0.006), but it was similar at End (*P* = 0.347). During the 20%MVC task the VL rmsEMG activity progressively increased throughout the task (*P* < 0.001), whereas it progressively declined throughout the 70%MVC task (*P* < 0.001).

### Corticospinal Responses throughout Exercise

**Figure 4.**
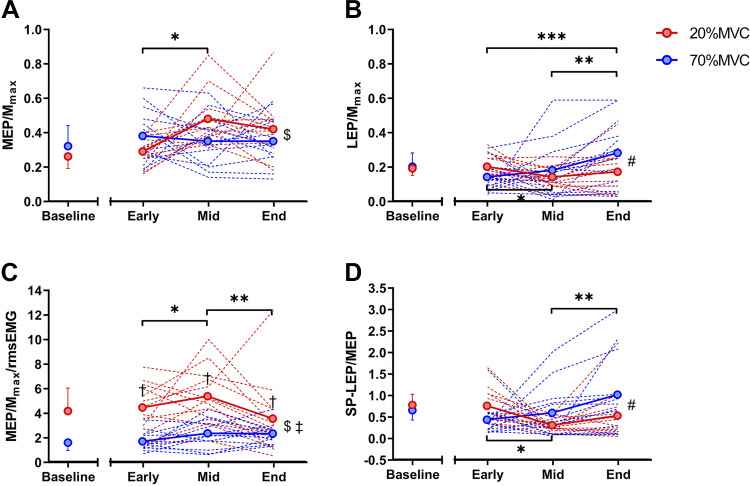
Corticospinal response during both fatiguing tasks: motor evoked potentials normalized to the maximal motor response (MEP/M_max_, *A*), lumbar evoked potentials elicited in the silent period (SP) after transcranial magnetic stimulation (TMS) normalized to the maximal motor response (LEP/M_max_, *B*), MEP/M_max_ normalized to the background root mean square electromyography activity ([MEP/M_max_]/rmsEMG, *C*), and SP-LEPs normalized to MEPs (SP-LEP/MEP, *D*) throughout the 20% maximal voluntary contraction (MVC) (in red) and 70%MVC (in blue) tasks. ‡Main effect of condition (*P* < 0.001). $Main effect of time (*P* ≤ 0.05). #Condition × time interaction (*P* ≤ 0.042). †Significantly different from 20%MVC or 70%MVC (*P* < 0.001). *Significantly different from Early to Mid (*P* < 0.05); **significantly different from Mid to End (*P* < 0.05); ***significantly different from Early to End (*P* < 0.05). Values are means ± SD for 13 participants.

Corticospinal excitability (MEP/M_max_) did not differ between tasks (*P* = 0.251) but demonstrated an effect over time (*P* = 0.028; Fig. 4*A*). When normalized to rmsEMG ([MEP/M_max_]/rmsEMG), corticospinal excitability was different between tasks (condition × time interaction; *P* < 0.001) and over time (*P* ≤ 0.039). More precisely, [MEP/M_max_]/rmsEMG during 20%MVC was higher compared with 70%MVC at Early, Mid, and End (*P* < 0.001; Fig. 4*C*). In addition, corticospinal excitability increased from Early to Mid in both tasks (*P* ≤ 0.038) and declined at End (*P* ≤ 0.035). Spinal excitability (LEP/M_max_; Fig. 4*B*) responded differently between tasks (condition × time interaction; *P* = 0.042). Specifically, spinal excitability decreased from Early to Mid during the 20%MVC task (*P* = 0.012) and increased from Early to End during the 70%MVC task (*P* ≤ 0.042; Fig. 4*B*). When spinal excitability was normalized to the MEP response (SP-LEP/MEP; Fig. 4*D*), a similar trend was observed (condition × time interaction; *P* = 0.047), showing overall a similar response between tasks (*P* ≥ 0.096). Spinal excitability decreased from Early to Mid during the 20%MVC task (*P* = 0.002) and increased throughout the 70%MVC task from Mid to End (*P* = 0.032).

### Electromyographic Response during Trapezoidal Ramps

VL rmsEMG of short- and long-duration ramps during the steady-state phase was significantly higher after the fatiguing tasks (*P* ≤ 0.001), without any differences between conditions (*P* ≥ 0.557) (see Supplemental Table S1).

### Motor Unit Decomposition

For long-duration ramps, nine participants were included in the analysis as it was not possible to extract reliable motor unit responses in four participants. A total of 283 motor units for coherence analysis were included. More specifically, 140 motor units in the 20% MVC task and 143 motor units in the 70%MVC task were found. The mean number of motor units included for each participant was 8 ± 3 and 8 ± 2 for the 20%MVC and 70%MVC tasks, respectively.

For short-duration ramps, 10 participants were included in the analysis as it was not possible to extract reliable motor unit responses in 3 participants. A total of 282 motor units from the short-plateau ramps for motor unit properties were included in the analysis. More specifically, 130 motor units in the 20% MVC task and 153 motor units in the 70%MVC task were identified. The mean ± SD number of motor units identified for each participant was 6 ± 2 and 7 ± 3 for the 20%MVC and 70%MVC tasks, respectively.

### Motor Unit Responses

**Figure 5.**
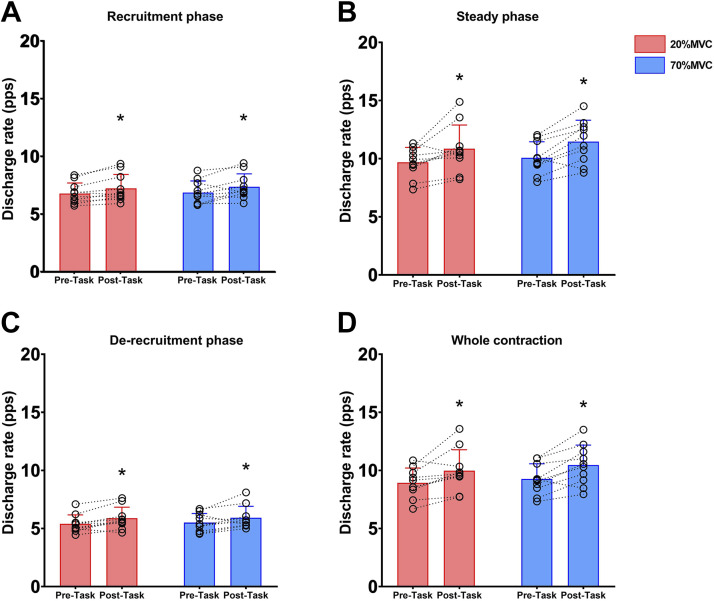
Motor unit discharge rate before and after both fatiguing tasks. Bar plots represent the average with data points representing individual data for motor unit discharge rate assessed during the recruitment phase (*A*), during the steady phase (*B*), during the derecruitment phase (*C*), and across the whole ramp (*D*) for both tasks measured at Pre-task and Post-task. MVC, maximal voluntary contraction. *Significantly different at Post-task (*P* < 0.05). Values are means ± SD for 10 participants.

Motor unit firing rate increased in all phases of the trapezoidal ramps during the 20%MVC ramps after performance of both the 20% and 70%MVC tasks (time effect; all *P* ≤ 0.043), without any difference between conditions (all *P* ≥ 0.178) and without significant condition × time interaction (all *P* ≥ 0.375). Results are illustrated in Fig. 5.

**Figure 6.**
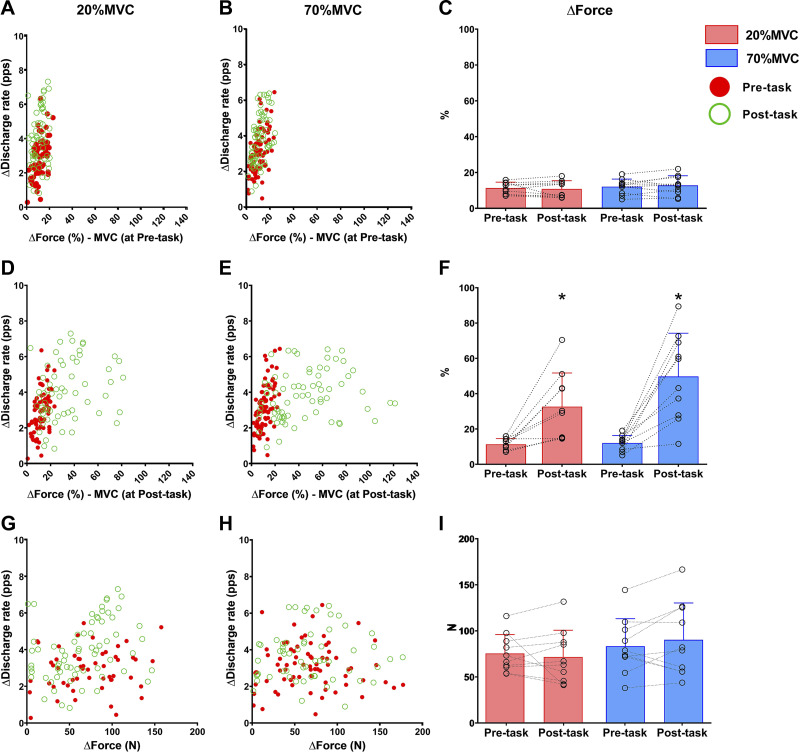
Estimated neural drive-to-muscle gain. *A* and *B*: scatterplots represent the association between the change in voluntary force as % of the maximal voluntary contraction (MVC) performed at Pre-task (*x*-axis, ΔForce) and change in motor unit discharge rate during the steady phase of the isometric ramp contractions (*y*-axis, ΔDischarge rate) in both tasks. *C*: bar plot shows the average ΔForce. *D* and *E*: scatterplots represent the association between the change in voluntary force as % of the MVC performed at Post-task (*x*-axis, ΔForce) and change in motor unit discharge rate during the steady phase of the isometric ramp contractions (*y*-axis, ΔDischarge rate) in both tasks. *F*: bar plot shows the average ΔForce. *G* and *H*: scatterplots represent the association between the change in voluntary force as % of the MVC (*x*-axis, ΔForce) and change in motor unit discharge rate during the steady phase of the isometric ramp contractions (*y*-axis, ΔDischarge rate) in both tasks. *I*: bar plot shows the average ΔForce. *Significantly different at Post-task (*P* < 0.05). Values are means ± SD for 10 participants.

Input-output gain for ΔForce–ΔDR and input-output gain for ΔForce_MVCpre-task_ and input-output did not change from Pre-task to Post-task (time effect; all *P* ≥ 0.684), with no difference between conditions (all *P* ≥ 0.116) and without condition × time interaction (all *P* ≥ 0.349). The input-output gain for ΔForce_MVCcorr_ –ΔDR increased at Post-task (time effect; *P* < 0.001), with no difference between conditions (main effect, *P* = 0.084; interaction effect, *P* = 0.086). Results are illustrated in Fig. 6.

The recruitment threshold and derecruitment threshold did not change from Pre-task to Post-task (time effect; all *P* ≥ 0.295), with no difference between conditions (all *P* ≥ 0.216) and without condition × time interaction (all *P* ≥ 0.396). Regarding motor unit coherence (see Supplemental Table S2), no changes were evident (*P* = 0.050, *P* = 0.099, *P* = 0.135, *P* = 0.763, and *P* = 0.889, respectively, for α, β, δ, γ, and total area; see Supplemental Table S3).

## DISCUSSION

The present study combined for the first time neurostimulation techniques and HDsEMG to investigate the neural adjustments contributing to decrements in neuromuscular function and alterations in motor units within the vastus lateralis muscle caused by fatiguing tasks performed at different intensities in healthy male participants. High-intensity exercise elicited a greater decline in neuromuscular function, whereas cortical and spinal responses increased through both tasks in a disparate manner. Motor unit behavior was altered similarly in both trials, with discharge rates increasing by ∼13% after exercise. The combination of neurostimulation and HDsEMG used in this study provided novel insight into how central and peripheral adjustments contribute to alterations in motor unit behavior in a large locomotor muscle group. The data suggest that a compensatory increase in spinal excitability occurs in response to contractile dysfunction, which partially contributes to an increase in motor unit discharge rate.

### Variations of Central and Peripheral Function after Fatiguing Exercise

Large decrements in neuromuscular function were elicited by both 20%MVC and 70%MVC protocols in this study, with 60% and 70% reductions in MVC after the respective trials. The cause of this loss in volitional force was multifactorial, with large decreases in Q_tw.pot_ and VA reduced by a third concurrently observed. These alterations support previous literature demonstrating a combination of contractile dysfunction and central nervous system impairment after sustained high (30–33)- and low (5, 6, 34, 35)-intensity contractions.

The 70%MVC trial elicited greater contractile dysfunction compared with the 20%MVC trial. Both intensities likely reside above the participants’ “critical torque” [∼15% MVC during sustained contractions, (3, 36)], a transition threshold above which metabolic stress is considered uncontrollable (37). The additional contractile dysfunction in the 70%MVC trial is likely a result of the blood flow occlusion that occurs during contractions above 50–60% MVC (38), the consequent augmented accumulation of intramuscular metabolites (39), and depletion of high-energy intramuscular phosphate stores (3, 40, 41). The rate at which these processes occurred was greater in the 70%MVC trial, which based on the decline in force produced (Fig. 2) was equivalent to a maximal voluntary effort for the majority of the trial. In addition, it was potentially due to the increased demand of contraction as well as the ischemia due to occlusion.

During fatiguing contractions, corticospinal excitability is thought to increase as a compensatory mechanism for contractile dysfunction within the musculature and reduced neural drive from the motor cortex (42, 43). However, when measured at task failure, robust evidence of changes in MEP amplitudes with fatigue is not clear, with previous studies showing no change (44–46), an increase (47–50), or a decrease (51, 52). This heterogeneity could be mediated by the intensity of exercise, with divergent responses observed after contractions above and below critical torque (53). Indeed, as both trials in the present study likely took place above critical torque, it is perhaps unsurprising that no trial × time interaction effect was observed for the change in corticospinal excitability with fatigue.

Subcortical stimulation of the spinal cord is commonly used to assess changes in corticospinal input to the motoneuron pool (54, 55). This study employed lumbar stimulation to investigate lower limb motoneuronal excitability and demonstrated increased excitability throughout the 70%MVC trial (Fig. 4). Similar single-joint studies in the lower limbs have demonstrated a decrease in spinal excitability during fatiguing contractions (24, 56), although lumbar stimulation might be less sensitive to change compared with thoracic stimulation (56). The sensitivity of the motoneuronal pool to synaptic input is thought to be reduced by multiple factors (12) including the activation-induced alterations of intrinsic motoneuron properties (24), the withdrawal of facilitatory group Ia afferent input to the motoneuron pool (57), and inhibitory influence of exaggerated group III/IV afferent feedback (58). It is therefore possible that the increased spinal excitability in the 70%MVC trial could have been counterbalanced by exaggerated inhibitory feedback from III/IV afferent neurons, resulting in equivalent changes in MU discharge rates between trials. However, one limitation of the neurostimulation techniques employed in the present study is that it is not possible to determine the relative contributions of these distinct neurophysiological processes to the fatigue-induced changes in LEP/M_max_.

### Variations in Motor Unit Behavior and Coherence after Fatiguing Exercise

Impairments in force generation capacity of exercising muscle are in part caused by alterations of the motor unit properties. Previous authors reported a reduction in motor unit firing rate during and after fatiguing tasks (59–62). In contrast, the present study showed higher discharge rate of motor units after both tasks. This finding is not exclusive to this study, as an acute increase in firing rate was previously reported during knee extensor tasks (14, 15, 63, 64).

Previous studies showed that acute adjustments during low-intensity fatiguing contractions do not progress linearly over time but rather change as the fatiguing task progresses. More precisely, Adam and De Luca (63) proposed that the increase in firing rates toward the end of the fatiguing task at 20%MVC was concomitant with recruitment of new motor units. A similar biphasic trend where discharge rate of motor units initially decreased and increased toward the end of the fatiguing task was also observed by Martinez-Valdes et al. (14) at 30% MVC and by Valenčič et al. (16) during sustained and intermittent tasks at 20%, 30%, and 50% MVC of the knee extensors. Similarly, Contessa et al. (64) showed that firing rates increased toward the end of repeated fatiguing contractions at 30% MVC alongside the recruitment of new motor units. Other studies reporting a monotonic decrease in firing rate involved high-intensity contractions at 50% MVC (59) and from 10% to 64% MVC (65) that contrast to what was observed after the 70%MVC trial.

Previous authors proposed that an increase of descending signals from cortical areas to the motoneuron pool is required to compensate for the decline in force generation capacity of the muscle during fatiguing tasks (4, 64, 66, 67). Our study showed that spinal excitability increased at the end of exercise (Fig. 4, *B* and *D*), thus supporting previous authors that the increase in motor unit discharge rate is likely caused by neural adjustments required to compensate for changes in contractile properties (42, 43). It must be acknowledged that a number of studies have shown that the firing rate of motor units decreased despite the increased excitation to the motoneuron pool (68–70). However, we cannot provide a precise explanation for our contrasting results. Our findings might be caused by the different exercise tasks and/or methodological differences (see *Limitations and Methodological Concerns* for more details).

Another aim of this study was to verify whether exercise intensity would have elicited different changes in motor unit properties. The two exercise intensities caused disparate responses in spinal excitability/responsiveness and surface EMG behavior throughout the tasks, along with exaggerated losses in maximal voluntary force and contractile function at task failure. Thus, an effect on motor unit behavior was likely expected. However, no difference in motor unit properties between the two tasks was found after task failure. To the best of our knowledge, only a limited number of studies have investigated this specific aspect in exercise until task failure. Our findings are in line with Castronovo and colleagues (15), where no difference in the motor unit firing rate after a sustained task at three different intensities of the tibialis anterior muscle were reported (20%, 50%, and 75% of MVC). Similarly, Conwit and colleagues (71) did not show any difference in firing rate at task failure of the vastus medialis between two contraction intensities (10% and 30% MVC). In our study, the lack of effect of exercise intensity on motor unit properties might rely on the fact that motor units were evaluated at low-intensity contraction ramps (i.e., 20% MVC). High-threshold motor units are recruited at higher contraction intensities. Therefore, the low-intensity contraction ramps did not allow us to monitor the whole spectrum of motor unit properties, thus possibly preventing the observation of behavioral changes of higher-threshold motor units. It should be considered that both tasks are well known to elicit significant impairments in force production and motor control, thus making it difficult for participants to optimally perform high-intensity contraction ramps and so negatively affecting any information regarding high-threshold motor units. Additionally, it should be noted that the force produced at the end of the two tasks was similar (∼20%), thus influencing the behavior and affecting the population of MUs available to produce the required task.

Recruitment and derecruitment threshold of motor units were not affected by the fatiguing tasks or by contraction intensity. A reduction in recruitment threshold is proposed as an additional compensatory mechanism when muscle fatigue develops (72, 73). This reduction might be peripherally affected by changes in the mechanical and metabolic properties of the muscle (74, 75). However, the current knowledge on recruitment threshold during and after fatiguing contractions remains equivocal. Previous authors proposed that these changes are not uniform across the motoneuron pool, as they might depend upon on activation timing and mechanical properties (59, 72). Studies involving the vastus lateralis reported a decrease in recruitment threshold of motor units without changes in recruitment order (63, 64), with similar findings observed in first dorsal interosseous (76). Conversely, other studies did not report any change in recruitment and derecruitment threshold of the first dorsal interossei and abductor hallucis (59, 68, 69) or reported an increase in recruitment threshold only for early recruited motor units of the abductor pollicis (72). These observations suggest that motor unit adjustments during fatiguing contractions depend on muscle-specific properties (i.e., percentage of low- and high-threshold motor units and upper limit recruitment) and therefore the findings obtained from individual muscles cannot be generalized. It should be acknowledged that recruitment and derecruitment thresholds depend also on the changes of the individual motor unit twitch forces. The lack of change in recruitment threshold may indicate that low-threshold units (those involved and analyzed during low 20%MVC ramps) did not change their force twitch properties. Other factors, such as contraction duration (repeated contractions vs. sustained contraction until failure) and contraction intensity, might explain the differences in results across studies.

Synchronization between the firings of active motor units is assumed to increase during fatiguing contractions, but this was not observed in the present study. Synchronization of motor unit firing can be observed in specific frequency ranges of δ (1–4 Hz), α (8–12 Hz), β (15–30 Hz), and γ (30–60 Hz) frequency bands. Beta-band coherence is of particular interest because it reflects information regarding cortical and subcortical oscillatory processes (77). No changes in motor unit coherence were found at task failure or between conditions. Our findings are partially in line with Hwang and colleagues (78), who reported an increase in alpha-band width only. In contrast, McManus and colleagues (60) observed a significant increase in δ, α, and β frequency bands after a fatiguing task of the first dorsal interosseous muscle at 30% MVC. Castronovo and colleagues (15) showed an increase only for δ and α frequency bands of the tibialis anterior muscle after 20% and 50% MVC, respectively, which was explained to be caused by the higher level of activity of the supraspinal and spinal pathways (i.e., increased input to the motoneuronal pool). This contrasts with what was found in the present work, and such discrepancies might highlight muscle- or task-specific changes in motor unit coherence.

### Limitations and Methodological Concerns

There are some limitations within the present study that should be discussed. Firstly, we were unable to extract motor units in a reliable way during the fatiguing tasks, and therefore it was not possible to firmly establish potential cause-effect relationships between corticospinal response and motor unit behavior. In addition, the process of motor unit extraction during fatiguing tasks remains a significant challenge and requires further optimization. Fatiguing tasks are well known to induce substantial changes in the EMG spectrum, thus negatively affecting the identification of motor units by decomposition algorithms (79).

A second limitation is that it was not possible to track individual motor units before and after the fatiguing tasks or across both experimental sessions. This approach introduced by Martinez-Valdes et al. (14) is based on tracking across time the motor unit spike times, which is based on the spatiotemporal filters built and tracked interactively by the blind source separation. However, we were not able to track individual motor units, most probably as a consequence of the profound changes in motor unit action potential shape induced by the fatiguing tasks or movement artifacts in the lower limb muscles. Therefore, our findings are based on groups of different motor units across sessions rather than acute changes of individual motor units.

It should also be acknowledged that despite large locomotor muscles being a better representative of many daily life activities, they still represent a challenge for HDsEMG decomposition algorithms. A lower number of motor units identified in the vastus lateralis compared with other muscles (e.g., first dorsal interosseous or tibialis anterior muscles) has been previously reported with the decomposition method (80). The differences in muscle architecture, amount of tissue between muscle and EMG electrodes, physiological noise, as well as interindividual differences caused a great deal of heterogeneity in the number of identified motor units for each participant. Therefore, our conclusions are based on a relatively small proportion of the total number of active motor units; perhaps a larger number of arrays might help to capture a better picture given the size of the muscle. Notwithstanding, these data do provide novel insights into the behavior of these locomotor muscles under fatiguing conditions. These methodological aspects have been discussed by previous authors, as they represent a limiting factor to understanding acute changes of motor unit properties in fatigue state (79, 80).

This study employed two distinct tasks to discern how motor unit firing characteristics responded to high- and low-intensity contractions. By nature, these two tasks resulted in different levels of voluntary EMG at the onset of and during contraction. Whereas MEPs normalized to background EMG show divergent responses during high- and low-intensity contractions (Fig. 3), one cannot be completely confident that the differing levels of neural drive between the two tasks were not the mechanisms underpinning the divergent response rather than intrinsic properties of the corticospinal tract. Alternate approaches to studying evoked responses during fatiguing contractions have been employed to counter this [i.e., constant-EMG tasks (24, 81)]; however, as the present study aimed to manipulate the surface EMG response (Fig. 3*B*) to study the underpinning changes in motor unit recruitment and firing properties (Fig. 5), this approach was not possible in the present study.

Finally, this study only tested male participants, and given the known sex differences in exercise-induced fatigue the data cannot be generalized to females (82). Females are well known to have a lower number of detectable MUs compared with males. A recent work by Taylor et al. (83) showed that, on average, males yielded nearly twice the number of MUs as females when measured in nonfatigued muscle. Further investigation is required to study potential sex differences in motor unit behavior (84) but also to improve the yield of motor units that are detectable by HDsEMG in female populations (83).

### Conclusions

In summary, this study further demonstrated that fatiguing exercise at different intensities produces distinct changes in neuromuscular functions. High-intensity exercise induced a greater reduction in maximal force production and impairments in contractile function whereas central fatigue was not affected by exercise intensity. During exercise, high-intensity contractions impaired the corticospinal excitability compared to low-intensity contractions whereas spinal excitability increased during the high-intensity contractions. Motor unit discharge rate increased after fatiguing exercise, but it was not affected by exercise intensity. Recruitment, derecruitment threshold, and coherence properties of motor units were also not affected by exercise. Based on our findings, mechanisms occurring at spinal level are likely the main contributors to the changes in motor unit behavior during fatiguing tasks.

## DATA AVAILABILITY

Data will be made available upon reasonable request.

## SUPPLEMENTAL DATA

10.6084/m9.figshare.25057607. Supplemental Tables S1–S3: https://doi.org/10.6084/m9.figshare.25057607.

## GRANTS

The authors received no specific funding for this work.

## DISCLOSURES

No conflicts of interest, financial or otherwise, are declared by the authors.

## AUTHOR CONTRIBUTIONS

L.A., A.D., S.G., K.T., and G.H. conceived and designed research; L.A., S.G., K.T., P.A., E.A., and G.H. performed experiments; L.A., S.G., K.T., and P.A. analyzed data; L.A., S.G., and P.A. interpreted results of experiments; L.A. prepared figures; L.A., A.D., S.G., P.A., and G.H. drafted manuscript; L.A., A.D., S.G., K.T., P.A., E.A., D.F., and G.H. edited and revised manuscript; L.A., A.D., S.G., K.T., P.A., E.A., D.F., and G.H. approved final version of manuscript.
